# Assessment of the autonomic response to a high dose CO_2_ inhalation challenge based on heart rate variability and skin conductance in a healthy population

**DOI:** 10.1016/j.ibneur.2025.10.014

**Published:** 2025-10-27

**Authors:** Hanna-Dalia Laar, Hanna M.T. Carlman, Robert J. Brummer, Julia Rode

**Affiliations:** a School of Medical Sciences, Faculty of Medicine and Health, & Nutrition-Gut-Brain Interactions Research Centre, Örebro University, Örebro, Sweden; b School of Health Sciences, Faculty of Medicine and Healt, Örebro University, Örebro, Sweden

**Keywords:** Autonomic arousal, Electrodermal activity, Electrocardiography, Hypercapnia, Panic (disorder)

## Abstract

Stress induction tests such as carbon dioxide (CO_2_) inhalation challenges, are often used in research of anxiety and panic disorders. Physiological parameters of the autonomic response, e.g., heart rate variability (HRV) and skin conductance (SC), are often measured alongside questionnaires for evaluation. Previous studies have shown varied results on CO_2_ inhalation-induced physiological reactivity and further knowledge is of interest. The aim of this study was to explore the effects of a 35 % CO_2_ inhalation test on HRV frequencies and SC, in healthy subjects; and to set those into relation with subjective psychological ratings. In this single-blinded, repeated measures study, healthy subjects underwent a 35 % CO_2_ inhalation challenge, whereof the first and third double vital capacity inhalations were with normal air and the second with 35 % CO_2_. HRV (low and high frequency) and SC (as electrodermal activity (EDA)) were measured throughout. CO_2_ inhalation resulted in a significant increase of HRV’s high and very high frequencies compared to the first air inhalation (median difference + 0.0001633; + 0.0000348) and of HRV’s very high and very low frequencies compared to the last air inhalation (+ 0.0000321; + 0.0000154). Mean and maximum SC increased significantly during the CO_2_ inhalation compared to both air inhalations (mean difference to first air inhalation + 1.151; + 1.964; to last air inhalation + 0.5751; +1.484), but also between the separate air inhalations (+ 0.5754; + 0.4799). The HRV results indicate increased parasympathetic activity, while the SC results indicate increased sympathetic activity during CO_2_ inhalation. SC only minorly correlated with provoked panic symptoms (EDA minimum to VAS minimum r = −0.559, and not between any other EDA and VAS/PSL measure). While those results seem contradictory, this study confirms that a 35 % CO_2_ inhalation challenge in young healthy adults, provokes a physiological as well as psychological reaction.

## Highlights

• Double vital capacity inhalations with 35 % CO_2_ induced distinct autonomic response.
• 35 % CO₂ inhalation evoked both physiological and psychological responses.
• High frequency HRV changes suggest increased parasympathetic activity due to CO_2_.
• Skin conductance changes suggest increased sympathetic activity during CO_2_ challenge.

## 1 Introduction

The autonomic nervous system (ANS) consists of two branches, the sympathetic nervous system (SNS) and the parasympathetic nervous system (PNS). These branches show some simultaneous activity and are balanced by parallel control in several target organs, such as the heart and the gut; i.e. increased sympathetic activity implies decreased parasympathetic activity and vice versa. The heart is one of the target organs that has dual control from both branches (Karemaker, 2017). Contrary, sweat release is only controlled by the SNS (Macefield et al., 2013).

The vagal nerves are major components of the PNS, which is mostly active during ‘rest and recovery’ (Karemaker, 2017). The SNS is mainly active during psychological, and physical (environmental or physiological) stress. Stress has been described as a condition of threatened homeostasis (Bali and Jaggi, 2015). The stress response includes biological (ANS and neuroendocrine system), psychological (emotion and cognition) and behavioural (fight or flight) adjustments to manage various danger and stress (Bali and Jaggi, 2015, Karemaker, 2017).

The ANS activity can be measured indirectly with peripheral markers such as heart rate (HR), heart rate variability (HRV) and sweat release (Macefield et al., 2013). HRV is the variation of time intervals between heartbeats and can be assessed by Fourier analysis of HR. Increased low-frequency (LF) HRV (0.04–0.15 Hz) has been associated with both increased PNS and SNS activity, and is regulated by baroreflex activity. Increased high-frequency (HF) HRV (0.15–0.40 Hz), based on more variations associated with lower HR, has been primarily linked with increased parasympathetic stimulation of the heart (Bali and Jaggi, 2015). A significant increase in LF HRV and a simultaneous decrease in HF HRV, have been observed during stress (Kuehl et al., 2015, McDuff et al., 2014). Another parameter of autonomic activity is skin conductance (SC), which measures levels of sweat release (increased sweating increases SC), and therefore exclusively measures the SNS activity (Macefield et al., 2013). Results from previous studies show increased SC during hypercapnic stress induction tests (Bystritsky et al., 2000, Pappens et al., 2012, Poma et al., 2005).

Stress induction tests, including carbon dioxide (CO_2_) inhalation challenges, are often used in research of panic disorder and other anxiety disorders to study pathophysiology and various treatments (Amaral et al., 2013, Bali and Jaggi, 2015, Bystritsky et al., 2000, Griez et al., 2007). Stress induction tests are also important for studying the physiological and pathophysiological changes during stress (Bali and Jaggi, 2015). The majority of studies that use stress induction tests include physiological parameters as an objective measure, in addition to subjective questionnaires, for the evaluation of the tests’ responses (Bailey et al., 2005, Blechert et al., 2010, Diaper et al., 2012, Griez et al., 2007, Leibold et al., 2013, Pappens et al., 2012, Poma et al., 2005).

The CO_2_ inhalation test is a safe and non-invasive method to induce short-lived anxiety or panic attacks, and physiological stress responses (Amaral et al., 2013, Argyropoulos et al., 2002, Bali and Jaggi, 2015, Griez et al., 2007, Van Den Hout and Griez, 1984). In the past, different dosages of CO_2_ as well as different inhalation techniques (single- vs double-vital capacity) have been used, consequently leading to the observation of different effects. The systematic review by Amaral et al., summarising CO_2_ challenge tests in panic disorder, showed that the majority of studies (conducted during 1984–2012) used the method with the 35 % CO_2_ protocol (Amaral et al., 2013). This comparably higher dose regime of 35 % CO_2_ double-vital capacity inhalation has been shown to evoke a stronger panic attack-like state (assessed with Panic Symptom List (PSL) and electronic visual analogous scale (VAS) for affect) as well as increased blood pressure, compared to lower dose regimes (i.e., 9–17.5 % CO_2_) (Griez et al., 2007, Leibold et al., 2013). Hence the effects of CO_2_ inhalation is thought to be dose-dependent (Griez et al., 2007, Leibold et al., 2013). Leibold et al. (2013) showed that the effects on HR variation (which is similar to HRV, but less specific) were also dose-dependent to CO_2_, and correlated with symptom ratings (assessed by VAS). It is believed that low dose (7.5 %) regimes of CO_2_ inhalations produce little physiological effects in healthy subjects (Bailey et al., 2005). Moreover, it has been shown that healthy subjects more seldomly respond with a panic attack (based on symptom assessments) during CO_2_ inhalation tests (13 %, 20 %, 35 %), than patients with diagnosed panic disorder (Forsyth et al., 2000, Caldirola et al., 1997, Coryell and Arndt, 1999). Thus, when studying subjects without panic or anxiety disorders, considering the dose-dependent effects of CO_2_ and the lower frequency of responders in the general population, it is favourable to use methods employing higher CO_2_ doses, and possibly double-dose vital capacity inhalations.

The purpose of this study was to observe the effects of a 35 % CO_2_ inhalation challenge on HRV and SC, as measures of autonomic activity, which was hypothesised to increase across all measures, in healthy subjects. The study, moreover, aimed to assess whether HRV and SC are equally well suited to evaluate the autonomic response to CO_2_ inhalation; and to set those objective measures into relation with subjective measures such as PSL-IV and VAS.

## 2 Material and methods

### 2.1 Design

This was a single-blinded study in healthy subjects. HRV and SC were measured to assess ANS activity during CO_2_ inhalation, compared to air inhalations before and after the CO_2_ inhalation. The CO_2_ challenge was part of another study focused on the interventional effects of probiotics (i.e. a nutritional supplement composed of living microorganisms beneficial for human health) (NCT03615651), with additional study activities conducted at Örebro University. The CO_2_ inhalation challenge was part of the phenotypic characterisation of the study population – of which the subjective ratings, but not objective ANS measurements, have been published (Rode et al., 2022).

Original ethical approval was given on 10th January 2018 by the Ethical Review Board of Uppsala, Sweden (registration number: 2017/398 A and B). The study was conducted in 2018 according to Good Clinical Practice and in accordance with the Helsinki Declaration of 1975 and its revisions. All participants signed an informed consent before any study-related activity.

### 2.2 Participants

22 subjects, included if aged 18–65 years, with final mean age 24.2 ± 3.4 years, performed the CO_2_ inhalation challenge. Exclusion criteria (for details see Supplementary Table S1) were designed to recruit a self-reported healthy population, and ensure the safety of conducting the CO_2_ inhalation test and the other examinations (reported in Rode et al. (2022)). In brief, relevant exclusion criteria for the CO_2_ inhalation test were asthma, epilepsy, pregnancy, breastfeeding, (history of) cerebral bleeding, a major psychiatric or somatic disease diagnosis (such as diabetes or cardiovascular diseases, amongst others).

### 2.3 Procedure of CO_2_ inhalation challenge

The participants were fasting for 12 h (this was needed for other measures in the previously reported study e.g., blood drawing) and given a light breakfast on site at Örebro University prior to the CO_2_ inhalation challenge. Information about the test procedure, safety, eventual sensations evoked and that they could terminate the experiment at any point, was given before start. A baseline recording period of on average 50 min for HRV and SC measures allowed for calibration and washout of potential artifacts due to previous events. Practice inhalations were conducted beforehand.

The CO_2_ inhalation challenge consisted of three consecutive double vital capacity inhalations. The first and third inhalations contained normal air, and the second contained a medical gas mixture of 35 % CO_2_ and 65 % oxygen. The participants were informed that only one of the double vital capacity inhalations would contain the CO_2_ gas mixture, but not which of the three. At the end of each double vital capacity inhalation, the participants held their breath for five seconds. A double vital capacity inhalation approach was chosen in order to ensure exposure to the medical gas mixture instead of solely inhaling the remainder air in the connecting tube which was at the shortest length practically possible.

Participants breathed through a nasal-oral face mask that was connected to a bag with the gas mixture. Delivery of the gas was turned on with a valve by the test conductor during the second inhalation. All inhalations were conducted identically, except the gas delivery valve was turned off during the first and third inhalations; instead, the test conductor simulated turning on the valve. The participants could not see the valve. They followed oral instructions by the researcher standing behind them. No gas tank was used which would have ensured participants’ impression of anyhow inhaling a gas while the valve was turned off.

In order to ensure proper conduction of the experiment, a practice inhalation with exact same setup, but normal air, and without the test conductor pretending to be turning on the valve, was conducted prior to the three consecutive experimental inhalations. The number of three exposures and their order as air – CO_2_ – air was selected to allow a single-blinded design that was not counterbalanced in order to standardise anticipation effects to the extend possible. Furthermore, the last inhalation, which was normal air, in this way, could be used to control and validate the first inhalation, which was also normal air.

### 2.4 Subjective perception ratings

For one minute following each exposure, participants were asked to rate their experience of fear and discomfort continuously using several consecutive visual analogous scales (VAS), of which mean, minimum and maximum rating as well as area under the curve (AUC) were calculated. Thereafter, participants rated the Panic Symptom List IV (PSL). This part of the dataset has previously been published in Rode et al. (2022).

### 2.5 ANS measurement with HRV and SC

HRV and SC were measured with the BioPac system MP150 with ECG100C and EDA100C (BioPac Inc., Goleta, CA, USA) during the CO_2_ inhalation test.

HRV measuring is described in more detail in Edebol Carlman et al. (2022). In summary, a continuous electrocardiograph (ECG) was recorded and then a custom-designed peak detector algorithm was used to determine the R-R intervals for the assessment of HRV.

For SC measuring, two electrodes (EL507) were placed on cleaned dry skin, on the index and middle finger (palmar side) of the non-dominant hand, to record electrodermal activity (EDA) with a sample rate of 1000 Hz (resampled to 65 Hz for analysis).

AcqKnowledge 5.0 (BioPac Inc., Goleta, CA, USA) was used for processing the ECG and EDA waveforms, also for analysing HRV in the frequency domains and the quantified autonomic balance, respectively, as well as obtaining SC values. The ECG and EDA data were analysed in time-blocks (one time-block for each double vital capacity inhalation), which were labelled during recording. A time-block started approximately 20 s before the double vital capacity inhalation, included the time of the CO_2_ or air inhalation itself (naturally individual duration per participant) and ended 65 s after, and hence lasted approximately 2 min totally. The experiment continued as soon as a time-block was completed, which led to approximately 30 s between time-blocks (i.e., exposure 1, 2, 3). This experimental setup combines the assessment of HRV and SC in all response phases: anticipation, inhalation and subsequent reaction of/to 35 % CO_2_, compared to normal air.

Data extracted from the ECG recordings were HRV frequencies (very LF (VLF) at < 0.04 Hz, LF at 0.04–0.15 Hz, HF at 0.15–0.40 Hz, and very HF (VHF) at 0.40–3.00 Hz) and autonomic activity proportions (AcqKnowledge calculation based on e.g., the power in the high HRV frequencies for the parasympathetic proportion, i.e.*,* the sum of HF and VHF HRV divided by the sum of all frequency values). The minimum, maximum and mean values of SC were extracted from each time block, from the EDA recordings.

Signal artifacts in the ECG and EDA waveforms were visually identified and modified with standard procedures e.g., connecting endpoints and band-pass filtering for ECG, median smoothing and low pass filtering for EDA.

Cut-offs were set for exclusion of low-quality data with relevant artifacts, that resulted in unidentifiable QRS-complexes, amongst others. Cut-off criteria for exclusion were: 1) exclusion of one time-block if ≥ 20 % of the waveform in a block had relevant artifacts, 2) total exclusion of a participants’ data if two of the three blocks met the first exclusion criterium.

### 2.6 Excluded data

One participants’ data (ECG and EDA) was excluded from analysis because the time-blocks could not be identified. ECG data from one additional participant was excluded because it met the second exclusion criterium. Two participants’ time-blocks were excluded from the ECG data because they met the first exclusion criterium. This resulted in 21 values available for analysis from each inhalation time-block from the EDA data. Remaining values for analysis from the ECG data were 20 values in the first inhalation time-block, and 19 values in the second and third inhalation time-blocks.

### 2.7 Statistical analysis

Statistical analysis was performed with GraphPad Prism (versions 9.1.0 and 10.2.2., GraphPad Software Inc., Boston, MA, USA). All datasets were tested for normal distribution using the Shapiro-Wilk test.

Testing for outliers with the ROUT method (aggression of removal Q = 1 %) was conducted because there was some uncertainty about one participants’ EDA wave quality. For method consistency, the HRV and autonomic activity proportions data were also tested for outliers, although there was no suspicion of poor ECG quality (after exclusion of low-quality data according to the above-described cut-offs). Outliers were not removed from subsequent analyses, instead abundance of outliers was treated as a result.

Median imputation was done to the HRV and autonomic activity data, if a time-block but not an entire participant was excluded due to the criteria mentioned above, to substitute excluded values so the statistical analysis could be conducted.

To assess the effect of the CO_2_ inhalation in comparison to normal air on the various outcome measures separately, normally distributed data (SC) was analysed using repeated-measures Analysis of Variance (RM ANOVA) and *posthoc* Tukey’s corrected multiple comparisons test. Non-normally distributed data (HRV and autonomic activity, PSL, VAS) was analysed with Friedman test and *posthoc* Dunns’ corrected multiple comparisons test. The level of significance was set to p < 0.05 for analysis of both the normally and non-normally distributed data.

To assess whether the various outcomes give proper representation of each other, Spearman correlations of CO_2_-triggered reactions were based on delta values of exposure 2 and 1 (∆Exp2−Exp1), hence CO_2_ inhalation and first air inhalation, of the various EDA measures (EDA mean, minimum and maximum), HRV measures (VHF, HF, LF and VLF HRV), as well as the subjective ratings PSL (PSL total score) and VAS (VAS mean, minimum, maximum, AUC) previously reported in Rode et al. (2022). Here, descriptive p-values are reported.

#### 2.7.1 Outliers

No outliers were detected in the EDA and the autonomic activity proportions data. A total of 22 outliers were detected in the HRV data, presented in Supplementary Table S2. In short, most outliers were found in data from the CO_2_ inhalation (n = 11). The outliers were not excluded and were interpreted as a result.

## 3 Results

### 3.1 CO_2_ inhalations’ effect on HRV

VHF, HF and VLF HRV values, but not LF HRV values differed significantly between the exposures (respectively: p = 0.0002, F = 16.90; p = 0.0078, F = 9.70; p = 0.0408, F = 6.40; and p = 0.7047, F = 0.70; df = 2 for all; Friedman tests).

**Fig. 1:**
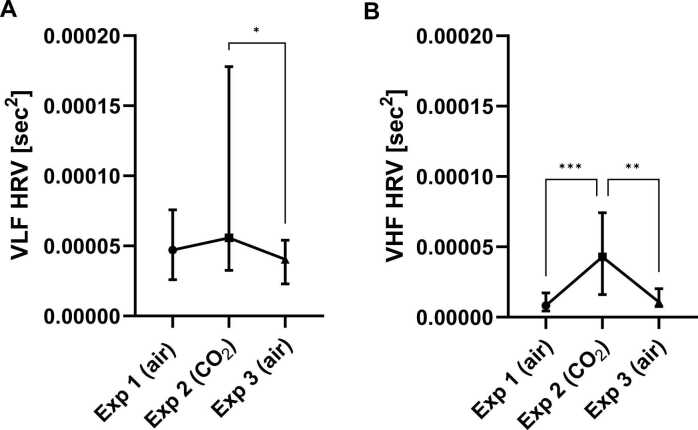
Effect of CO_2_ inhalation on heart rate variability (HRV). (**A**) Very low frequency (VLF) HRV and (**B**) very high frequency (VHF) HRV measured in sec^2^, for each exposure (Exp). Symbol presents median (connected with line). Error bar presents interquartile range. *p < 0.05, **p < 0.01, ***p < 0.001, ****p < 0.0001, from *posthoc* Dunn’s multiple comparisons. Abbreviation: sec, seconds.

**Fig. 2:**
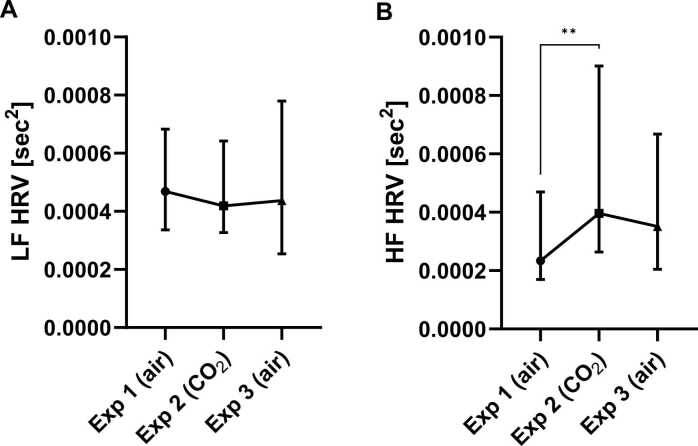
Effect of CO_2_ inhalation on heart rate variability (HRV). (**A**) Low frequency (LF) HRV and (**B**) high frequency (HF) HRV measured in sec^2^ for each exposure (Exp). Symbol presents median (connected with line). Error bar presents interquartile range. *p < 0.05, **p < 0.01, ***p < 0.001, ****p < 0.0001 from *posthoc* Dunn’s multiple comparisons. Abbreviations: sec, seconds.

Dunn’s multiple comparisons test showed that CO_2_ inhalation (exposure 2) statistically significantly increased the HRV frequencies, compared to normal air inhalations (exposure 1 or 3). The significant differences were found between VLF HRV values of exposure 2 and 3 (+ 0.0000154, p = 0.0342), VHF HRV values of exposure 1 and 2, as well as exposure 2 and 3 (+ 0.0000348, p = 0.0008; + 0.0000321, p = 0.0015) and HF HRV values between exposure 1 and 2 (+ 0.0001633, p = 0.0080) (Fig. 1, Fig. 2, Supplementary Table S3).

### 3.2 CO_2_ inhalations’ effect on the autonomic activity proportions

A significant difference in autonomic activity proportions between the exposures was found with the Friedman test (p = 0.0106, F = 9.10, df = 2). As described in the methods section, the parasympathetic activity proportion is calculated from the power in the VHF and HF frequency bands.

**Fig. 3:**
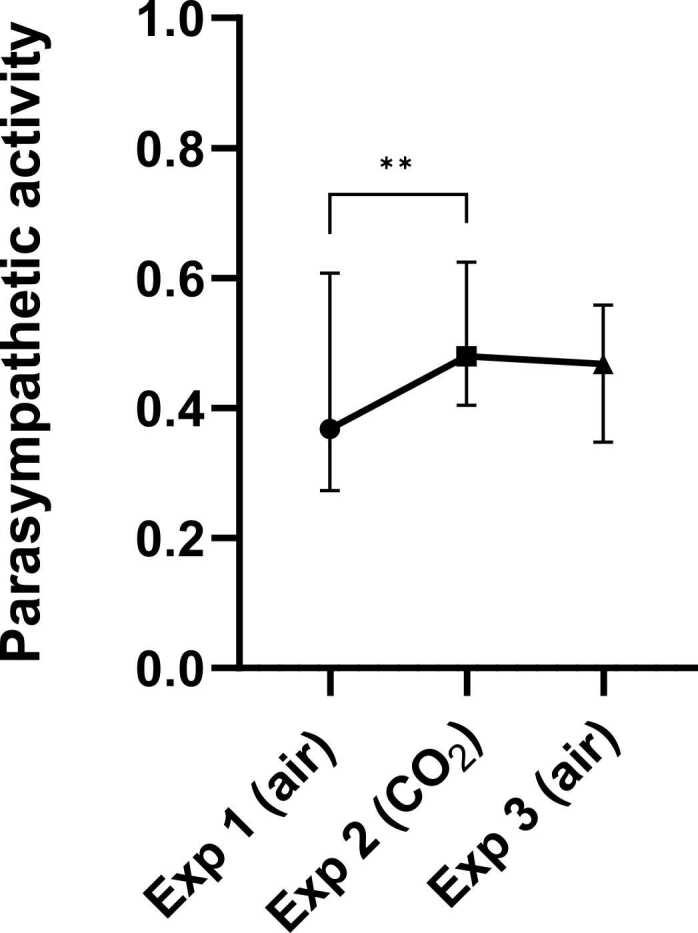
Effect of CO_2_ inhalation on the parasympathetic activity proportion; as relative proportion of high frequencies (VHF + HF) heart rate variability (HRV) from all frequencies (VHF + HF + LF + VLF). Symbol presents median (connected with line). Error bar presents interquartile range. *p < 0.05, **p < 0.01, ***p < 0.001, ****p < 0.0001 from *posthoc* Dunn’s multiple comparisons. Abbreviations: Exp, exposure; VHF, very high frequency; HF*,* high frequency; LF, low frequency; VLF, very low frequency.

Dunn’s multiple comparisons test showed that the parasympathetic activity significantly increased from the first air inhalation (exposure 1) to the CO_2_ inhalation (exposure 2) (+ 0.1122, p = 0.0080) (Fig. 3, Supplementary Table S3).

### 3.3 CO_2_ inhalations’ effect on SC

Statistically significant differences between the exposures were found in EDA maximum, minimum and mean data, with RM ANOVA analysis (respectively: p < 0.0001, F = 28.16; p = 0.0001, F = 18.24; p < 0.0001, F = 25.50; df = 2, 20 for all).

**Fig. 4:**
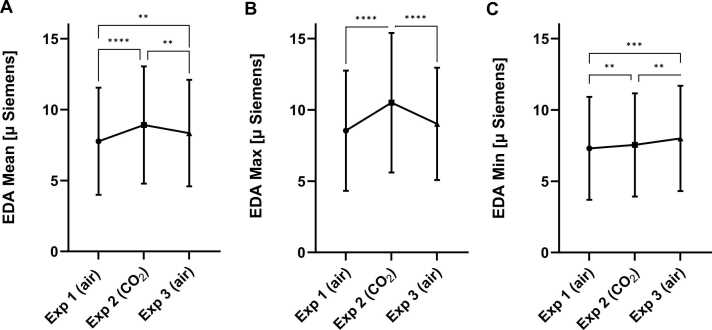
Effect of CO_2_ inhalation on skin conductance (SC). Electrodermal activity (EDA) (**A**) Mean, (**B**) Maximum (Max) and (**C**) Minimum (Min) values measured in μSiemens for each exposure (Exp). Symbol presents mean (connected with line), error bar presents standard deviation. *p < 0.05, **p < 0.01, ***p < 0.001, ****p < 0.0001 from *posthoc* Tukey’s multiple comparisons.

Tukey’s multiple comparisons test on the mean, maximum and minimum values from each exposure’s time-block showed a statistically significant increase in SC in all EDA measures, when comparing CO_2_ (exposure 2) with air inhalations (exposure 1 (EDA mean + 1.151, p < 0.0001; EDA max + 1.964, p < 0.0001; EDA min + 0.2423, p = 0.0013) and exposure 3 (EDA mean + 0.5751, p = 0.0025; EDA max + 1.484, p < 0.0001; EDA min − 0.4586, p = 0.0060)). A statistically significant difference was also found when comparing the first and last exposures (both normal air inhalations) in the EDA mean (−0.5754, p = 0.0012) and minimum values (−0.7008, p = 0.0003); but not for the EDA maximum values (Fig. 4, Supplementary Table S4).

### 3.4 CO_2_ inhalations’ effect on panic symptoms

**Fig. 5:**
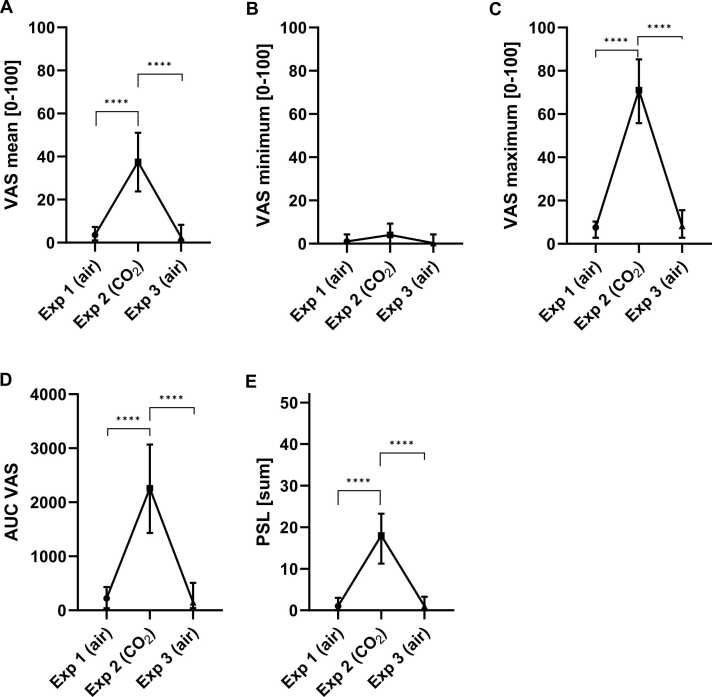
Effect of CO_2_ inhalation on subjective ratings. Visual analogous scale (VAS) (**A**) Mean, (**B**) Minimum and (**C**) Maximum values measured on a scale from 0 to 100, (**D**) their area under the curve (AUC) over multiple ratings during 60 s, and (**E**) total score of the Panic Symptom List (PSL-IV) for each exposure (Exp). Symbol presents median (connected with line), error bar presents interquartile range. *p < 0.05, **p < 0.01, ***p < 0.001, ****p < 0.0001 from *posthoc* Dunn’s multiple comparisons.

As reported previously (Rode et al., 2022), inhalation of 35 % of CO_2_ caused panic symptoms according to PSL-IV and multiple VAS during the duration of 60 s post inhalation. Statistically significant differences between the exposures were found in total PSL, mean and maximum VAS ratings as well as VAS AUC with the Friedman test (respectively: p < 0.0001, F = 36.99; p = 0.0001, F = 30.80; p < 0.0001, F = 32.10; p < 0.0001, F = 30.51; df = 2 for all) (Fig. 5, Supplementary Table S5). Only the minimum VAS rating did not differ significantly between the inhalations.

Dunn’s multiple comparisons test showed that the ratings significantly increased from the first air inhalation (exposure 1) to the CO_2_ inhalation (exposure 2), as well as significantly decreased again to the last air inhalation (exposure 3), for all p < 0.0001. None of the ratings differed significantly between the two air inhalations (exposure 1 vs 3).

Few participants (4/22) did not experience symptoms based on PSL-IV for panic symptom evaluation and VAS to assess level of fear and discomfort.

### 3.5 Correlation of CO_2_-triggered HRV and SC, and with subjective ratings

CO_2_-triggered (∆Exp2−Exp1) EDA minimum significantly correlated moderately negative with HF HRV (r = −0.474, p = 0.0405) and VAS minimum (r = −0.559, p = 0.0085) (for full correlation matrix please refer to Supplementary Table S6). No other EDA measure correlated significantly with any HRV measure, nor did any other EDA or HRV measure correlate significantly with any other subjective rating (PSL total score or the various VAS measures).

## 4 Discussion

This study aimed to explore and refine the methodological approaches to measure autonomic response to the 35 % CO_2_ inhalation challenge in healthy, young adults.

We hypothesised that all outcome measures (HRV, EDA) would significantly increase in response to the CO_2_ inhalation compared to air inhalation. Yet, it was of interest whether both outcome measures are equally well suited to evaluate the autonomic response and whether the various outcomes give proper representation of each other, or can be represented by subjective ratings.

Overall, CO_2_ inhalation led to an increase in HRV (especially high frequencies) as well as SC (all EDA measures), compared to normal air inhalation, in young healthy subjects. Only few of the various measures correlated.

Specifically, we observed that the HF and VHF HRV increased significantly, during CO_2_ inhalation, compared to the preceding air inhalation, while no effects on the low frequencies (LF and VLF HRV) were seen. This is in line with previous studies where HF components of HRV increased with hypercapnia (Brown et al., 2007; Brown and Howden, 2008; Sasano et al., 2002). The origins of the HRV frequency components are still somewhat controversial (Brown et al., 2007). Many studies state that the LF HRV is exclusively connected to the sympathetic cardiac stimulation, however without clear supporting evidence for this (Macefield et al., 2013). LF HRV has also been associated with both the SNS and PNS although its interpretation has been questioned (Reyes del Paso et al., 2013), whilst HF HRV has mostly been associated with only parasympathetic activity (Bali and Jaggi, 2015; Brown et al., 2007; Macefield et al., 2013).

Accordingly, McDuff et al. (2014) observed an increased LF HRV, as well as increased respiratory frequency (RF), during stress induction with a mental arithmetic task. Also, Kuehl et al. (2015) observed a decrease in HF HRV, and an increased RF, during mental stress induction by a challenging reaction/time task. The conclusion from their study was that stress-induced RF increase, and parasympathetic activity decrease, are responsible for the mental stress effects on HF HRV (Kuehl et al., 2015). As opposed to those mental/cognitive stressors, the 35 % CO_2_ inhalation test is considered a four-dimensional stressor, provoking responses in four domains: physiological (e.g. HRV, SC), psychological (e.g. panic sensations), behavioural (e.g. conscious respiration changes), cognitive (e.g. attention) (Bali and Jaggi, 2015), while other stress tests mainly activate the hypothalamic-pituitary-adrenal (HPA) axis and modulate the serotonergic system. Hence the difference in respiration rate in the present study (controlled deep breaths), and the study by Kuehl et al. (2015) and McDuff et al. (2014) (free breathing; increased RF), might explain the differences in the HRV results during the stress tests; considering that all the tests induce mental stress. Instructed deep breathing has been shown to lead to increased HF HRV alone (Grossman, 2024), however, respiratory effects could not be controlled for in the presented dataset. Yet, any effect of the instructed deep breathing should be comparable in the CO_2_ and air inhalations.

A number of outliers were detected in the HRV data, and the majority were found in the second exposure, indicating that the participants were more affected during the CO_2_ inhalations. Although visual inspection of the ECG waves did not indicate poor electrode connection, potentially more caution with the experiment conduction e.g., controlling electrode placement and connection, as well as monitoring the participants’ movement level such as due to breathing, could be beneficial to minimise the number of outliers in future studies. However, the participants were not moving more than what could have been expected in such situation, and to impose more restrictions in this already controlled situation would have been unnatural and might well have disturbed the participants’ initial relaxation and normal breathing as well as added anticipatory stress interfering with the experiment.

The observed increase in the HRV’s high frequencies (HF and VHF HRV) in this study, may be interpreted as a simultaneous increase of PNS and a decrease of SNS activity, considering the counterbalancing parallel regulation of the heart by the ANS (Karemaker, 2017). The HRV results from this study can be thought of as somewhat contradictory because sympathetic activity is thought to increase during “normal” stress and anxiety, which are intended to be induced directly and indirectly by the CO_2_ inhalation test (Bali and Jaggi, 2015). Yet, the results may be explainable if the CO_2_ inhalation triggered an overstimulation of the PNS leading to a vasovagal reaction characterised by very low blood pressure and heart rate while sweating (Edwards et al., 2004, Ocon et al., 2011). Possibly, the CO_2_ inhalation challenge resulted in an acute induction of high stress followed by a compensation with increased PNS activity.

A physiological factor that has been shown to affect HRV is increased partial pressure of CO_2_ (PaCO_2_), which stimulates carotid chemoreceptors with an excitatory effect on vagal preganglionic neurons to the heart in the expiration phase of breathing (Sasano et al., 2002). Therefore, the increased PNS activity during the CO_2_ inhalations in this study, may have resulted from increased PaCO_2_. Further analyses could not give additional insights on the influence of PaCO_2_ on the results. The small number of participants who did not experience panic symptoms (n = 4) defined by subjective ratings (as reported by Rode et al. (2022) did not allow for stratified analyses of responders versus non-responders. Also, CO_2_-triggered reactions using the various SC measures, HRV measures as well as the subjective ratings PSL and VAS correlated only minimally, indicating that the correlation might not be linear and rather bi-phasic, if present at all. While the significant increase of total PSL-IV scores and average, maximum and AUC of multiple VAS ratings during CO_2_ inhalation, compared to the both air inhalations, but not between the two air inhalations, confirms that the intended effect of the CO_2_ inhalation test (panic induction) was obtained (Rode et al., 2022), our results indicate that the method for measuring ANS activity with HRV might not be suitable during CO_2_ inhalation tests.

As expected, we found that SC increased significantly during the CO_2_ inhalation compared to air inhalations, which reflects increased sympathetic stimulation during CO_2_ inhalation (Bali and Jaggi, 2015, Macefield et al., 2013). This is in line with previous studies where SC increased during CO_2_ inhalation challenges (Bystritsky et al., 2000, Pappens et al., 2012, Poma et al., 2005), which further validates this method for evaluating the ANS activity during CO_2_ inhalation challenges. Yet, a statistically significant difference was also found between the first and last air inhalations. Considering the slow responses of SC (Macefield et al., 2013), this difference between the air inhalations might result from not enough recovery time for the SC to reset to baseline level, after the CO_2_ inhalation.

Studies with laboratory experiments infer the advantage of maintaining controlled conditions. Yet, controlled conditions also include limitations; the results and conclusions cannot necessarily be generalised. Although the experimental inhalations were single-blinded, the CO_2_ gas mixture may be distinguished by taste and smell. Therefore, the participants may have concluded that the last inhalation, hence after CO_2_ inhalation, would be normal air. Thus, in theory, the anticipated anxiety, that participants may have had prior to CO_2_ inhalation, could have decreased for the last inhalation, while the anticipated anxiety may have been relatively equal and hence more comparable before the first and second inhalations. This last inhalation may be used as a validation for the comparison of the first two inhalations, such as that decreased HRV or SC in the last inhalation verify the increase from first air inhalation to CO_2_ inhalation. Also, the last inhalation was important to allow for the single-blinded conduction of the test, else the participants would – after the first normal air inhalation – conclude that the second inhalation must contain CO_2_. The application of a gas tank also for normal air inhalations could have partly compensated for the altered anticipation, since it would have supported participants’ impression of anyhow inhaling a gas. Nevertheless, subjective ratings (PSL, VAS) did not differ between the two air inhalations, and in fact returned to the levels of exposure 1 at exposure 3 – hence, here no order effects were observed.

The setup of this experiment included the assessment of HRV and SC in response to anticipation, inhalation and subsequent reaction of/to 35 % CO_2_ compared to normal air. Notable, some of the assessed parameters may be largely indicative of one or some but not all of these response phases. For instance, the measurement of EDA minimum predominantly represents the anticipation phase. Please refer to Supplementary Fig. S7 for representative ECG and EDA curves. The separation of those response phases during analyses, would require a more exact reporting of every participant’s individual timing and duration of the double-vital capacity inhalation, than was possible in the conduction of this trial and data collection.

The sample size of this study may be perceived as rather small, yet partly compensated for by the repeated measurements within-subjects. Since the CO_2_ inhalation challenge was part of the phenotypic characterisation of the study population in the previously reported probiotic intervention study (Rode et al., 2022), no prior sample size calculation was performed specifically for this assessment. The study population consisted mostly of women (n = 16); a stratified subgroup analysis was not conducted due to the small number of male participants (n = 6), despite uncertainty if CO_2_ inhalation-induced physiological reactivity differs between the sexes, with various studies providing contradictory results (Gregor and Zvolensky, 2008, Kelly et al., 2006, Leibold et al., 2013, Nillni et al., 2012). Baseline ratings for psychological symptoms were below any clinical or subclinical cut-off (Rode et al., 2022), yet the majority of participants were susceptible to panic symptoms. The young age (24.2 ± 3.4 years) of the study population limits generalisability of the results. The behavioural vulnerability (measured with VAS for affect) to CO_2_ inhalations has been shown to be greater for younger compared to older subjects (18–31 vs 38–62 years of age) (Griez et al., 2007) which may be associated to the progressive decline of panic symptomatology in panic disorder patients and a lower prevalence of panic disorder in the older population (Krystal et al., 1992, Sheikh et al., 2004). In turn, however, due to the differences in prevalence and potentially pathophysiology, the knowledge value might be greater in studying younger subjects.

Our results may indicate that SC, but not HRV, might be suitable measures for autonomic responses, when conducting the CO_2_ inhalation test. We believe this study gives valuable information for choosing physiological measurements of stress when conducting CO_2_ inhalation tests.

Using a variety of assessment methods, this study confirms that a 35 % CO_2_ inhalation challenge in young healthy adults, provokes a physiological as well as psychological reaction, possibly indicating the nature of a healthy stress response.

## CRediT authorship contribution statement

**Hanna-Dalia Laar:** Writing – review & editing, Writing – original draft, Visualization, Methodology, Investigation, Formal analysis, Data curation. **Brummer Robert:** Writing – review & editing, Methodology, Investigation, Funding acquisition, Conceptualization. **Hanna Carlman:** Writing – review & editing, Project administration, Methodology, Investigation, Conceptualization. **Julia Rode:** Writing – review & editing, Writing – original draft, Validation, Supervision, Project administration, Methodology, Investigation, Formal analysis, Data curation, Conceptualization.

## Compliance with ethical standards

The CO_2_ challenge was part of another study (NCT03615651), with additional study activities conducted at Örebro University. Original ethical approval was given on 10th January 2018 by the Ethical Review Board of Uppsala, Sweden (registration number: 2017/398 A and B). The study was conducted in 2018 according to Good Clinical Practice and in accordance with the Helsinki Declaration of 1975 and its revisions. All participants signed an informed consent before any study-related activity.

## Funding source

This study was partly supported by the Swedish Knowledge Foundation (Grant no. 20150081).

## Conflicts of Interest

None of the authors has any conflict of interest to declare.

## Data Availability

The data are pseudonymised, and the key variable may not be destroyed. According to Swedish ethics regulations, the raw data cannot be shared without an approved ethics application from the National Swedish Ethics Authority. An ethical permit can only be obtained for research being conducted within Sweden. Data can be requested as a public document via forskningsdata@oru.se and will have to undergo a confidentiality assessment to assess what can be released.

## References

[bib1] Amaral J.M., Spadaro P.T., Pereira V.M., Silva A.C., Nardi A.E. (2013). The carbon dioxide challenge test in panic disorder: a systematic review of preclinical and clinical research. Rev. Bras. Psiquiatr..

[bib2] Argyropoulos S.V., Bailey J.E., Hood S.D., Kendrick A.H., Rich A.S., Laszlo G., Nash J.R., Lightman S.L., Nutt D.J. (2002). Inhalation of 35 % CO(2) results in activation of the HPA axis in healthy volunteers. Psychoneuroendocrinology.

[bib3] Bailey J.E., Argyropoulos S.V., Kendrick A.H., Nutt D.J. (2005). Behavioral and cardiovascular effects of 7.5 % CO2 in human volunteers. Depress. Anxiety.

[bib4] Bali A., Jaggi A.S. (2015). Clinical experimental stress studies: methods and assessment. Rev. Neurosci..

[bib5] Blechert J., Wilhelm F.H., Meuret A.E., Wilhelm E.M., Roth W.T. (2010). Respiratory, autonomic, and experiential responses to repeated inhalations of 20 % CO2 enriched air in panic disorder, social phobia, and healthy controls. Biol. Psychol..

[bib6] Brown S.J., Howden R. (2008). The effects of a respiratory acidosis on human heart rate variability. Adv. Exp. Med. Biol..

[bib7] Brown S.J., Mundel T., Brown J.A. (2007). Cardiac Vagal Control and Respiratory Sinus Arrhythmia during Hypercapnia in Humans. J. Physiol. Sci. JPS..

[bib8] Bystritsky A., Craske M., Maidenberg E., Vapnik T., Shapiro D. (2000). Autonomic reactivity of panic patients during a CO2 inhalation procedure. Depress. Anxiety.

[bib9] Caldirola D., Perna G., Arancio C., Bertani A., Bellodi L. (1997). The 35 % CO2 challenge test in patients with social phobia. Psychiatry Res..

[bib10] Coryell W., Arndt S. (1999). The 35 % CO2 inhalation procedure: test-retest reliability. Biol. Psychiatry.

[bib11] Diaper A., Nutt D.J., Munafò M.R., White J.L., Farmer E.W., Bailey J.E. (2012). The effects of 7.5 % carbon dioxide inhalation on task performance in healthy volunteers. J. Psychopharmacol. Oxf. Engl..

[bib12] Edebol Carlman H.M.T., Rode J., König J., Repsilber D., Hutchinson A.N., Thunberg P., Persson J., Kiselev A., Pruessner J.C., Brummer R.J. (2022). Probiotic mixture containing Lactobacillus helveticus, Bifidobacterium longum and Lactiplantibacillus plantarum affects brain responses to an arithmetic stress task in healthy subjects: a randomised clinical trial and proof-of-concept study. Nutrients.

[bib13] Edwards M.R., Benoit J., Schondorf R. (2004). Electrodermal activity in patientswith neurally mediated syncope. Clin. Auton. Res..

[bib14] Forsyth J.P., Eifert G.H., Canna M.A. (2000). Evoking analogue subtypes of panic attacks in a nonclinical population using carbon dioxide-enriched air. Behav. Res. Ther..

[bib15] Gregor K.L., Zvolensky M.J. (2008). Anxiety sensitivity and perceived control over anxiety-related events: Evaluating the singular and interactive effects in the prediction of anxious and fearful responding to bodily sensations. Behav. Res. Ther..

[bib16] Griez E.J., Colasanti A., van Diest R., Salamon E., Schruers K. (2007). Carbon dioxide inhalation induces dose-dependent and age-related negative affectivity. PLoS One.

[bib17] Grossman P. (2024). Respiratory sinus arrhythmia (RSA), vagal tone and biobehavioral integration: Beyond parasympathetic function. Biol. Psychol..

[bib18] Karemaker J.M. (2017). An introduction into autonomic nervous function. Physiol. Meas..

[bib19] Kelly M.M., Forsyth J.P., Karekla M. (2006). Sex differences in response to a panicogenic challenge procedure: an experimental evaluation of panic vulnerability in a non-clinical sample. Behav. Res. Ther..

[bib20] Krystal J.H., Leaf P.J., Bruce M.L., Charney D.S. (1992). Effects of age and alcoholism on the prevalence of panic disorder. Acta Psychiatr. Scand..

[bib21] Kuehl L.K., Deuter C.E., Richter S., Schulz A., Rüddel H., Schächinger H. (2015). Two separable mechanisms are responsible for mental stress effects on high frequency heart rate variability: an intra-individual approach in a healthy and a diabetic sample. Int. J. Psychophysiol. Off. J. Int. Organ. Psychophysiol..

[bib22] Leibold N.K., Viechtbauer W., Goossens L., De Cort K., Griez E.J., Myin-Germeys I., Steinbusch H.W.M., van den Hove D.L.A., Schruers K.R.J. (2013). Carbon dioxide inhalation as a human experimental model of panic: the relationship between emotions and cardiovascular physiology. Biol. Psychol..

[bib23] Macefield V.G., James C., Henderson L.A. (2013). Identification of sites of sympathetic outflow at rest and during emotional arousal: Concurrent recordings of sympathetic nerve activity and fMRI of the brain. Int. J. Psychophysiol..

[bib24] McDuff D., Gontarek S., Picard R. (2014). Annu. Int. Conf. IEEE Eng. Med. Biol. Soc. IEEE Eng. Med. Biol. Soc. Annu. Int. Conf. 2014.

[bib25] Nillni Y.I., Berenz E.C., Rohan K.J., Zvolensky M.J. (2012). Sex differences in panic-relevant responding to a 10 % carbon dioxide-enriched air biological challenge. J. Anxiety Disord..

[bib26] Ocon A.J., Medow M.S., Taneja I., Stewart J.M. (2011). Respiration drives phase synchronization between blood pressure and RR interval following loss of cardiovagal baroreflex during vasovagal syncope. Am. J. Physiol. Heart Circ. Physiol..

[bib27] Pappens M., De Peuter S., Vansteenwegen D., Van Den Bergh O., Van Diest I. (2012). Psychophysiological responses to CO2 inhalation. Int. J. Psychophysiol..

[bib28] Poma S.Z., Milleri S., Squassante L., Nucci G., Bani M., Perini G.I., Merlo-Pich E. (2005). Characterization of a 7 % carbon dioxide (CO2) inhalation paradigm to evoke anxiety symptoms in healthy subjects. J. Psychopharmacol. Oxf. Engl..

[bib29] Reyes del Paso G.A., Langewitz W., Mulder L.J.M., van Roon A., Duschek S. (2013). The utility of low frequency heart rate variability as an index of sympathetic cardiac tone: a review with emphasis on a reanalysis of previous studies. Psychophysiology.

[bib30] Rode J., Edebol Carlman H.M.T., König J., Hutchinson A.N., Thunberg P., Persson J., Brummer R.J. (2022). Multi-strain probiotic mixture affects brain morphology and resting state brain function in healthy subjects: an RCT. Cells.

[bib31] Sasano N., Vesely A.E., Hayano J., Sasano H., Somogyi R., Preiss D., Miyasaka K., Katsuya H., Iscoe S., Fisher J.A. (2002). Direct effect of Pa _CO 2_ on respiratory sinus arrhythmia in conscious humans. Am. J. Physiol. Heart Circ. Physiol..

[bib32] Sheikh J.I., Swales P.J., Carlson E.B., Lindley S.E. (2004). Aging and panic disorder: phenomenology, comorbidity, and risk factors. Am. J. Geriatr. Psychiatry Off. J. Am. Assoc. Geriatr. Psychiatry.

[bib33] Van Den Hout M.A., Griez E. (1984). Panic symptoms after inhalation of carbondioxide. Br. J. Psychiatry.

