# Supplementary material for: Assessment of the autonomic response to a high dose CO2 inhalation challenge based on heart rate variability and skin conductance in a healthy population
Source: IBRO Neurosci Rep. 2025 Oct 27;19:836–43. doi: 10.1016/j.ibneur.2025.10.014 (PMC12617614; doi:10.1016/j.ibneur.2025.10.014)
Supplement: Supplementary file 1 — Supplementary material [file mmc1.docx]

## **Supplementary information**

**Assessment of the autonomic response to a high dose CO_2_ inhalation challenge based on heart rate variability and skin conductance in a healthy population**

## Hanna-Dalia Laar^1^, Hanna MT Carlman^1^, Robert J Brummer^1*^ & Julia Rode^1, 2*^

1 School of Medical Sciences, Faculty of Medicine and Health, & Nutrition-Gut-Brain Interactions Research Centre, Örebro University, Örebro, Sweden

2 School of Health Sciences, Faculty of Medicine and Health, Örebro University, Örebro, Sweden

**Supplementary Table S1.** In- and exclusion criteria

| *Inclusion criteria* |
| --- |
| 1. 18-65 years of age |
| 1. Signed informed consent |
| *Exclusion criteria* |
| 1. Concurrent or recent (< twelve weeks) treatment with drugs affecting intestinal function or mood, e.g. antidepressants or antibiotics |
| 1. Concurrent or recent (< four weeks) use of nutritional supplements or herb products affecting intestinal function or mood (e.g. aloe vera, St. John’s Wort, fibres, prebiotics and probiotics) |
| 1. Diagnosis of major psychiatric or somatic disease |
| 1. Abuse of alcohol or drugs |
| 1. Recent (< four weeks) intake of proton pump inhibitors (e.g. omeprazol) |
| 1. Asthma |
| 1. Cardiovascular diseases |
| 1. Epilepsy |
| 1. Renal failure |
| 1. Cerebral bleeding or history of cerebral bleeding |
| 1. Allergy to latex |
| 1. Pregnancy (assessed by urine test) or breastfeeding |
| 1. Claustrophobia |
| 1. Smoking or using tobacco including snuff |
| 1. Inability to maintain exercise routine and dietary pattern during the study |
| 1. Consumption of more than six cups of coffee/caffeine-containing beverages per day |
| 1. Professional athlete |
| 1. Any contraindication to a magnetic resonance imaging (e.g. medical implant or device not compliant with magnetic resonance imaging) |
| 1. Recent (< three months) regular intake of systemic corticosteroids and anti-inflammatory medication (including non-steroidal anti-inflammatory drugs) |
| 1. Known allergy to milk or soy |
| 1. Any other reason the investigator felt the subject was not suitable for participation in the study |

**Supplementary Table S2.** Number of outliers in heart rate variability (HRV) data

|  | Exp 1 (air) | Exp 2 (CO_2_) | Exp 3 (air) |
| --- | --- | --- | --- |
| Very low frequency (VLF) HRV data | - | 4 | - |
| Low frequency (LF) HRV data | 1 | 2 | - |
| Very high frequency (VHF) HRV data | 3 | 2 | 2 |
| High frequency (HF) HRV data | 3 | 3 | 2 |
|  |  |  |  |
| Total | = 7 | = 11 | = 4 |

*Abbreviation:* Exp, Exposure

**Supplementary Table S3.** Test details for HRV

| Friedman test | | | *Posthoc* Dunn’s multiple comparisons | | | |
| --- | --- | --- | --- | --- | --- | --- |
| p-value | Friedman statistic | df | Comparison | Median difference | Adjusted p-value | Number of subjects |
| **VLF HRV** | | | | | | |
| 0.0408 | 6.40 | 2 | Exp 2 (CO_2_) vs. Exp 1 (air) | 0.0000087 | 0.6177 | 20 |
|  |  |  | Exp 3 (air) vs. Exp 1 (air) | -0.0000067 | 0.6177 | 20 |
|  |  |  | Exp 2 (CO_2_) vs. Exp 3 (air) | 0.0000154 | 0.0342 | 20 |
| **VHF HRV** | | | | | | |
| 0.0002 | 16.90 | 2 | Exp 2 (CO_2_) vs. Exp 1 (air) | 0.0000348 | 0.0008 | 20 |
|  |  |  | Exp 3 (air) vs. Exp 1 (air) | 0.0000026 | >0.9999 | 20 |
|  |  |  | Exp 2 (CO_2_) vs. Exp 3 (air) | 0.0000321 | 0.0015 | 20 |
| **LF HRV** | | | | | | |
| 0.7047 | 0.70 | 2 | Exp 2 (CO_2_) vs. Exp 1 (air) | -0.0000500 | >0.9999 | 20 |
|  |  |  | Exp 3 (air) vs. Exp 1 (air) | -0.0000318 | >0.9999 | 20 |
|  |  |  | Exp 2 (CO_2_) vs. Exp 3 (air) | -0.0000182 | >0.9999 | 20 |
| **HF HRV** | | | | | | |
| 0.0078 | 9.70 | 2 | Exp 2 (CO_2_) vs. Exp 1 (air) | 0.0001633 | 0.0080 | 20 |
|  |  |  | Exp 3 (air) vs. Exp 1 (air) | 0.0001182 | 0.0806 | 20 |
|  |  |  | Exp 2 (CO_2_) vs. Exp 3 (air) | 0.0000451 | >0.9999 | 20 |
| **Parasympathetic activity** | | | | | | |
| 0.0106 | 9.10 | 2 | Exp 2 (CO_2_) vs. Exp 1 (air) | 0.1122 | 0.0080 | 20 |
|  |  |  | Exp 3 (air) vs. Exp 1 (air) | 0.1005 | 0.2460 | 20 |
|  |  |  | Exp 2 (CO_2_) vs. Exp 3 (air) | 0.0117 | 0.6177 | 20 |

**Supplementary Table S4.** Test details for EDA

| Repeated measures ANOVA | | | *Posthoc* Tukey’s multiple comparisons | | | |
| --- | --- | --- | --- | --- | --- | --- |
| p-value | F | df1, df2 | Comparison | Mean difference | Adjusted p-value | Number of subjects |
| **EDA Mean** | | | | | | |
| <0.0001 | 25.50 | 2, 20 | Exp 2 (CO_2_) vs. Exp 1 (air) | 1.151 | <0.0001 | 21 |
|  |  |  | Exp 3 (air) vs. Exp 1 (air) | 0.5754 | 0.0012 | 21 |
|  |  |  | Exp 2 (CO_2_) vs. Exp 3 (air) | 0.5751 | 0.0025 | 21 |
| **EDA Max** | | | | | | |
| <0.0001 | 28.16 | 2, 20 | Exp 2 (CO_2_) vs. Exp 1 (air) | 1.964 | <0.0001 | 21 |
|  |  |  | Exp 3 (air) vs. Exp 1 (air) | 0.4799 | 0.0789 | 21 |
|  |  |  | Exp 2 (CO_2_) vs. Exp 3 (air) | 1.484 | <0.0001 | 21 |
| **EDA Min** | | | | | | |
| 0.0001 | 18.24 | 2, 20 | Exp 2 (CO_2_) vs. Exp 1 (air) | 0.2423 | 0.0013 | 21 |
|  |  |  | Exp 3 (air) vs. Exp 1 (air) | 0.7008 | 0.0003 | 21 |
|  |  |  | Exp 2 (CO_2_) vs. Exp 3 (air) | -0.4586 | 0.0060 | 21 |

**Supplementary Table S5.** Test details for subjective ratings

| Friedman test | | | *Posthoc* Dunn’s multiple comparisons | | | |
| --- | --- | --- | --- | --- | --- | --- |
| p-value | Friedman statistic | df | Comparison | Median difference | Adjusted p-value | Number of subjects |
| **PSL** | | | | | | |
| <0.0001 | 36.99 | 2 | Exp 2 (CO_2_) vs. Exp 1 (air) | 17.0 | <0.0001 | 22 |
|  |  |  | Exp 3 (air) vs. Exp 1 (air) | 0.0 | >0.9999 | 22 |
|  |  |  | Exp 2 (CO_2_) vs. Exp 3 (air) | 17.0 | <0.0001 | 22 |
| **VAS mean** | | | | | | |
| <0.0001 | 30.80 | 2 | Exp 2 (CO_2_) vs. Exp 1 (air) | 47.5 | <0.0001 | 22 |
|  |  |  | Exp 3 (air) vs. Exp 1 (air) | -1.0 | >0.9999 | 22 |
|  |  |  | Exp 2 (CO_2_) vs. Exp 3 (air) | 48.5 | <0.0001 | 22 |
| **VAS minimum** | | | | | | |
| 0.1956 | 3.26 | 2 | Exp 2 (CO_2_) vs. Exp 1 (air) | 3.0 | 0.5245 | 22 |
|  |  |  | Exp 3 (air) vs. Exp 1 (air) | -0.7 | >0.9999 | 22 |
|  |  |  | Exp 2 (CO_2_) vs. Exp 3 (air) | 3.7 | 0.7746 | 22 |
| **VAS maximum** | | | | | | |
| <0.0001 | 32.10 | 2 | Exp 2 (CO_2_) vs. Exp 1 (air) | 63.5 | <0.0001 | 22 |
|  |  |  | Exp 3 (air) vs. Exp 1 (air) | 0.8 | >0.9999 | 22 |
|  |  |  | Exp 2 (CO_2_) vs. Exp 3 (air) | 62.7 | <0.0001 | 22 |
| **VAS AUC** | | | | | | |
| <0.0001 | 30.51 | 2 | Exp 2 (CO_2_) vs. Exp 1 (air) | 2036.5 | <0.0001 | 22 |
|  |  |  | Exp 3 (air) vs. Exp 1 (air) | -73.5 | >0.9999 | 22 |
|  |  |  | Exp 2 (CO_2_) vs. Exp 3 (air) | 2110.0 | <0.0001 | 22 |

**Supplementary Table S6.** Correlation matrix

|  |  | EDA Min | EDA Max | EDA Mean | VLF HRV | LF HRV | VHF HRV | HF HRV | PSL | VAS max | VAS min | VAS AUC | VAS mean |
| --- | --- | --- | --- | --- | --- | --- | --- | --- | --- | --- | --- | --- | --- |
| EDA Min | p | x | 0.2731 | 0.0675 | 0.3710 | 0.8083 | 0.9829 | 0.0405 | 0.6660 | 0.2286 | 0.0085 | 0.7841 | 0.8100 |
|  | r | 1.000 | 0.251 | 0.406 | -0.218 | -0.060 | 0.005 | -0.474 | -0.100 | -0.274 | -0.559 | -0.064 | -0.056 |
| EDA Max | p |  | x | 0.0000 | 0.6628 | 0.2091 | 0.5233 | 0.9375 | 0.5031 | 0.7219 | 0.5477 | 0.8580 | 0.8449 |
|  | r |  | 1.000 | 0.940 | 0.107 | 0.302 | 0.156 | 0.019 | -0.155 | -0.083 | 0.139 | 0.042 | 0.045 |
| EDA Mean | p |  |  | x | 0.4286 | 0.4305 | 0.5715 | 0.6215 | 0.5821 | 0.4430 | 0.8041 | 0.8493 | 0.8405 |
|  | r |  |  | 1.000 | 0.193 | 0.192 | 0.139 | -0.121 | -0.127 | -0.177 | 0.058 | 0.044 | 0.047 |
| VLF HRV | p |  |  |  | x | 0.1000 | 0.7643 | 0.6114 | 0.4153 | 0.5980 | 0.7715 | 0.3072 | 0.3216 |
|  | r |  |  |  | 1.000 | 0.389 | 0.074 | 0.125 | 0.199 | 0.129 | 0.071 | 0.247 | 0.240 |
| LF HRV | p |  |  |  |  | x | 0.2366 | 0.0257 | 0.5344 | 0.7720 | 0.8149 | 0.5494 | 0.5278 |
|  | r |  |  |  |  | 1.000 | 0.285 | 0.510 | -0.152 | -0.071 | -0.058 | 0.147 | 0.154 |
| VHF HRV | p |  |  |  |  |  | x | 0.5617 | 0.5858 | 0.1105 | 0.3973 | 0.9829 | 0.9943 |
|  | r |  |  |  |  |  | 1.000 | 0.142 | 0.134 | -0.378 | 0.206 | 0.005 | 0.002 |
| HF HRV | p |  |  |  |  |  |  | x | 0.3703 | 0.2836 | 0.1917 | 0.2475 | 0.2444 |
|  | r |  |  |  |  |  |  | 1.000 | -0.218 | -0.259 | 0.313 | -0.279 | -0.281 |
| PSL | p |  |  |  |  |  |  |  | x | 0.0650 | 0.8137 | 0.0710 | 0.0710 |
|  | r |  |  |  |  |  |  |  | 1.000 | 0.400 | 0.053 | 0.392 | 0.392 |
| VAS max | p |  |  |  |  |  |  |  |  | x | 0.5956 | 0.0005 | 0.0005 |
|  | r |  |  |  |  |  |  |  |  | 1.000 | 0.120 | 0.679 | 0.684 |
| VAS min | p |  |  |  |  |  |  |  |  |  | x | 0.2075 | 0.2194 |
|  | r |  |  |  |  |  |  |  |  |  | 1.000 | 0.280 | 0.273 |
| VAS AUC | p |  |  |  |  |  |  |  |  |  |  | x | 0.0000 |
|  | r |  |  |  |  |  |  |  |  |  |  | 1.000 | 0.999 |
| VAS mean | p |  |  |  |  |  |  |  |  |  |  |  | x |
|  | r |  |  |  |  |  |  |  |  |  |  |  | 1.000 |


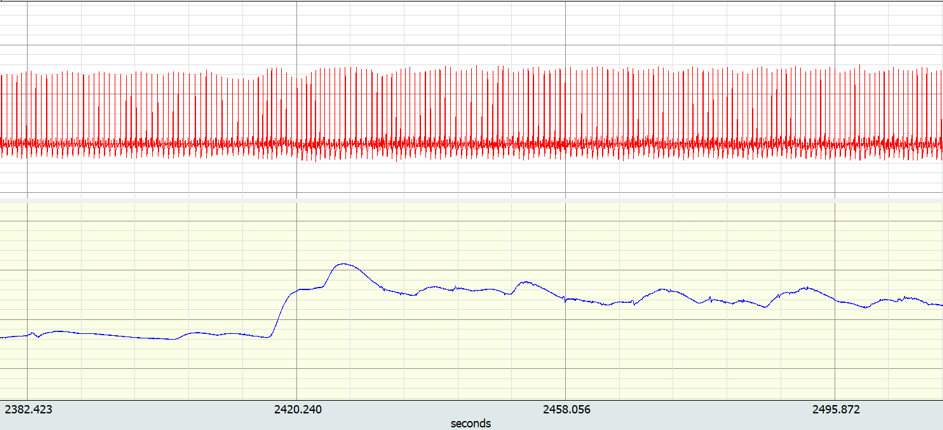


**Supplementary Figure S7.** Representative ECG (top) and EDA (bottom) curve from start to end of the CO_2_ inhalation, hence exposure 2. Image retrieved as screenshot from the analysis software, AcqKnowledge, based on this work’s data from one not-identifiable subject.
